# A profile of injuries within an Australian State Emergency Service Agency: a retrospective study

**DOI:** 10.1186/s12873-025-01429-z

**Published:** 2025-12-01

**Authors:** Graham Marvin, Elisa F. D. Canetti, Rob Orr, Ben Schram

**Affiliations:** Tactical Research Unit, Faculty of Health Sciences & Medicine, Bond University, Robina, QLD Australia

**Keywords:** Firefighter, Ambulance, Volunteer, Occupational safety

## Abstract

**Background:**

Injuries within the emergency services populations are unfortunately common. Effective injury reduction programs need to be designed based on the profile of common injuries. Therefore, this study aimed to profile the injuries suffered within a state Emergency Services Agency which comprised Ambulance, Fire and Rescue, Rural Fire, volunteer emergency State Emergency Services (SES), and the Communication Centre (CC).

**Methods:**

A retrospective cohort analysis was conducted on the entirety of an Australian State Emergency Service Agency injury database over a ten-year period (2012–2022). Records were extracted with details including (a) the total number; (b) the bodily site; (c) the nature; (d) and mechanism of injury. Total injuries were converted into injuries per 1000 full-time equivalent (FTE) years of service and incidence rate (IR) and ratios (IRR) were calculated per service.

**Results:**

In total, there were 2,703 physical injuries reported by the agency, with Ambulance Services sustaining over half the injuries (57.5%). The most common body site, nature of injury, and mechanism was the lower back (27.9%), soft tissue (59.7%), and body stressing (45.5%), respectively. Based on 1000 FTE years, SES had the highest IR of 2054.2 followed by Rural Fire (IR = 1295.1), Communications (IR = 753.7), Ambulance Services (IR = 566.3), and Fire and Rescue (IR = 183.7). State Emergency Services sustained the highest IRR of 3.64 [3.13–4.22] when compared to Ambulance Services. The age group most likely to be injured were 45–49 years of age (17%), with males suffering the majority (65%) of injuries.

**Conclusion:**

State Emergency Services present with injury rates above those of other emergency services personnel. These findings lay the groundwork for customised injury prevention strategies to promote better occupational safety across emergency service populations. Tailored injury prevention strategies may decrease subsequent time off due to injury.

## Introduction

Injuries among emergency first responders (EFR), such as ambulance officers and firefighters, are common [1, 2], occurring at a higher rate than in the general workforce [3]. Injuries in United States (US) ambulance personnel have increased from 86 per 1000 full-time equivalent working hours in 2017 [4] up to 345 per 1000 full-time working hours in 2022 [1]. In Australia, ambulance personnel injury rates averaged 80 serious cases per year for 1000 full-time working hours with paramedicine considered one of the most dangerous occupations [5]. Firefighters reportedly sustained 101 and 113 injuries per 1000 workers in Australia [6] and South Korea [7], respectively. More broadly in the US, over 350,000 firefighters were admitted to an emergency department suffering non-fatal injuries over the period 2003–2014 [8].

Many injuries to both ambulance and firefighting personnel in the US occur during patient care tasks [1, 8–10]. This may be due, in part, to ambulance and firefighters rating patient handling as a high physically-demanding task [11, 12]. While patient care related physical tasks do not always result in injury, injuries occurring during physical tasks are common. For example, ambulance report ‘body motion related’ [1], whereas firefighters report slips, trips, and falls as the primary mechanism of injury [2]. Potential reasons for these differences may be that ambulance rate stretcher manipulation [11], while firefighters rate patient rescue [12, 13], as the most physically demanding task. Understanding the how EFR task demands contribute to injury mechanism is vital for protecting not only ambulance and firefighters, but also EFR volunteers, who support these two services.

In Australia, volunteers are an integral part of EFR, allowing greater agency capacity and extending capabilities beyond paramedics and firefighters. One such example agency is the Australian State Emergency Services (SES). Volunteers working in the SES are vital in providing assistance during and after declared emergency disasters [14]. SES may provide support to firefighters but can also assist with storm and flood damage, traffic hazards, and road crash rescue [14]. The American Red Cross (ARC), a similar volunteer organisation, aims to prevent and alleviate the suffering of humans in emergencies by providing food, shelter, and support to individuals for a return to a life of routine [15]. Unfortunately, injury data on the aforementioned groups are sparce. While there are no known research studies profiling injuries in SES, the ARC has reported rates of 14 per 1000 responders for hurricanes from 2009 to 2012 [15]. However, no injury mechanisms have been stated by the ARC [15]. Thus, there is a limited understanding of the injuries in EFR volunteers.

Emergency first response is a complex network comprised of on-the-ground personnel, such as paramedics, firefighters (urban and rural), volunteers, and communication centre personnel. Communications centres role is multifaceted interactive task which requires continuously gathering and processing complex information to allow a swifter deployment of ground based EFR [16]. Despite the emergency situation not physically exposing the communications centre to danger, this type of EFR are still at risk of physical harm [17]. While limited, there is some evidence that states auditory injuries are the most common in this population [18, 19]. 

To ensure successful implementation of injury reduction programs, agencies must first understand the injury profile (and their contributing factors) of each EFR population. Thus, this study aimed to identify and compare injury profiles across five emergency response groups within a single Australian State Emergency Services Agency (ASESA) to inform tailored prevention strategies. It is hypothesised that there will be differences in injury rates, leading bodily sites, and mechanisms of injury but not natures of injury amongst the different EFR populations.

## Methods

A retrospective cohort analysis of the ASESA injury database was conducted over a 10-year period (2012–2022). This data were routinely collected within the agency’s database and was designed to capture and classify details of all incidents, hazards, near misses, and feedback of injuries within the agency. Data were extracted by a member of the ASESA who was familiar with the agency’s database and provided in a nonidentifiable format as an excel spreadsheet. Although police are typically used within the term ‘first responder’ [20], they were excluded from this study to focus on paramedics, firefighters, and those who perform functions in a volunteer capacity and emergency control centre [20].

Key variables of interest were the details of injuries for each service within the agency, which included an Ambulance Service, Fire and Rescue, Rural Fire, volunteer SES, and the Communications Centre. Data extracted included the anatomical location of injury (e.g., back, upper limb, lower limb), the nature of injury (e.g., soft tissue, lacerations), and the mechanism of injury (e.g., falls, body stressing), along with any differences between the various age groups and sex. Any data that was incomplete, related to illnesses, near misses, psychological injuries, or where data were classified as ‘no injury’ were excluded from analysis. Although there is a relationship between psychological injury and physical injury [6], physical injury was the primary variable.

As the data were non-identifiable, incomplete data could not be followed up. All classifications and definitions used within this study were based on the Type of Occurrence Classification System (TOOCS) [21] used within Australian injury databases. The TOOCS was developed to code details of worker’s injuries for compensation agencies [21]. Ethics approval for this project was granted by Bond University Human Research Committee (BS02192) and gatekeeper approvals provided by the Commissioner of the state emergency service.

Full-time equivalent (FTE) for each population was precalculated and given by the Justice and Community Safety Directorate in an Excel spreadsheet along with total headcount broken down by self-identified gender. Injury rates were calculated per 1000 FTE working hours to compare between populations. The incident risk ratio (IRR) was calculated by dividing the observed incidence of the injuries for each population compared to the highest injured population with 95% confidence intervals (95% CI). Each IRR was calculated as:

$$\:\widehat{\text{I}\text{R}}=\frac{{A}_{1}/{T}_{1}}{{A}_{0}/{T}_{0}}$$

$$\:SE\:[\text{ln}\left(\widehat{\text{I}\text{R}}\right)]=\:\left(\frac{1}{{A}_{1}}+\:\frac{1}{{A}_{0}}\right)1/2$$

$$(\:\widehat{I{R}_{L}},\:\widehat{I{R}_{U}}\:)\:=\:\exp\:\left\{\text{ln}\widehat{(\text{I}\text{R}})\right.\pm\:1.96\:x\:SE\:\left.[\text{ln}\left(\widehat{\text{I}\text{R}}\right)]\right\}$$

where *A*_1_ and *A*_0_ denote the number of injuries compared to the total number of people in each group, respectively, and *T*_1_ and *T*_0_ denote the corresponding total person-time in the two groups with a Wald-based approximate 95% CI for $\:\widehat{\text{I}\text{R}}$ [22].

Ambulance Services was used as the reference population for IRR, as this occupation is known for having the greatest number of injuries compared to the other EFR services [6]. Comparisons were then made between services, age groups, and gender.

## Results

A total of 4,470 events (including near misses, no injury, or psychological insults) were reported by the agency over the 10-year period from 2012 to 2022. After removal of the cases not involving physical injuries, a total of 2,703 injuries remained to be analysed. Of these, 1,570 occurred in the Ambulance Service (57.5%), 705 in the Fire and Rescue service (26%), 201 in the Rural Fire service (7.4%), 197 in the volunteer State Emergency Services (7.2%), and 31 in Communications Centre (1.1%).

Injuries to Fire and Rescue personnel were 30% less than those observed in Ambulance. Rural Fire suffered injuries by an increase of 129% compared to Ambulance. The most at-risk group for injuries was SES personnel, with nearly four times the injuries reported for Ambulance Services. Although Communications Centres sustained more injuries than Ambulance, this was not significant. Table 1 summaries the IRR of each service when compared to the Ambulance Service.

**Table 1:** Incident risk ratio for 10-year injury history

Population	10-year total injuries	10-year headcount	Full-time equivalent	Injuries per 1000 FTE years	IRR [95% CI]
Ambulance*	1570	2929	2772.1	566.3	1
Fire and Rescue	705	3888	3838	183.7	0.32 [0.30–0.35]
Rural Fire	201	160	155.2	1295.1	2.29 [1.97–2.64]
SES	197	100	95.6	2054.2	3.64 [3.13–4.22]
Coms Centre	30	43	39.8	753.7	1.33 [0.92–1.91]

* = Ambulance was used as the reference point to compare against all other populations. SES = State Emergency Service. Coms Center = Communications Centre

Of the 2,703 reported injuries, 1,759 (65%) were reported by male personnel while 863 (32%) were reported by female personnel. Gender was indeterminate/intersex/unspecified in the remaining 81 (3%) reported injuries. When injury rate was reviewed by age groups, the 45–49-year-old group sustained the most injuries with 462 (17%) followed by 40–44 years of age with 396 (13%) and 50–54 years of age with 313 (11.5%).

The upper limb (29%), posterior torso (23%), and the lower limb (17.6%) were the most commonly injured bodily locations among all the populations combined. Within each body region the lower back (27.9%), hand/wrist (22%), and the shoulder (13.8%) were the most common injured body parts (Table 2).

**Table 2:** Top three most common injuries by body part for each service

Population	Lower back *n* (%)	Hand/wrist *n* (%)	Shoulder *n* (%)	Knee *n* (%)	Head/Face *n* (%)	Foot/ankle *n* (%)	Ear *n* (%)
Ambulance Services	354 (22)	169 (10.7)	148 (9)	-	-	-	-
Fire and Rescue	81 (11.5)	108 (15.3)	-	89 (12.6)	-	-	-
Rural Fire	-	31 (15.4)	-	-	30 (14.9)	20 (10)	-
State Emergency Services	-	36 (18.2)	-	-	18 (9.1)	36 (18.2)	-
Communications Centre	3 (10)	3 (10)	-	-	-	-	6 (20)
Total of all services (%)	445 (16.4)	347 (12.8)	148 (5)	89 (3)	48 (1.7)	56 (2)	6 (0.2)

(n = total injuries for previous 10-years for that population, (%) is for overall percentage of injuries reported to that population)

All services, but Communications Centre, identified soft tissue as the most common nature of injury (60%). Other commonly reported nature of injury were contusions, bruises, or hematomas (6.7%), and superficial injuries (5.9%) as depicted in Table 3.

**Table 3:** Top three most common injury nature for each service

Population	Soft tissue *n* (%)	Contusions *n* (%)	Superficial *n* (%)	Lacerations *n* (%)	Deafness *n* (%)	Poisoning/toxic substances *n* (%)
Ambulance Services	1072 (68.2)	78 (4.9%)	75 (4.7)	-	-	-
Fire and Rescue	432 (61.2)	44 (6.2)	-	53 (7.5)	-	-
Rural Fire	46 (22.8)	18 (8.9)	35 (17.4)		-	-
State Emergency Services	67 (34)	31 (15.7)	13 (11.6)	13 (11.6)	-	-
Communications Centre	4 (13.3)	-	-	-	6 (20)	4 (13.3)
Total of all services (%)	1621 (60)	171 (6)	123 (4)	66 (2)	6 (0.2)	4 (0.1)

(n = total injuries for previous 10-years for that population, (%) is for overall percentage of injuries reported to that population)

The mechanism of injury identified between all agencies, but Rural Fire, were reported as body stressing (45.5%). Although being hit with a moving object was second most common (10.6%) hitting objects with body (10.4%) was reported by all but the Communications Centre as seen in Table 4.

**Table 4:** Top three most common mechanisms of injury for each service

Population	Body stressing *n* (%)	Being hit by moving object *n* (%)	Hitting objects with body *n* (%)	Slips, trips, falls *n* (%)	Chemicals and other substances *n* (%)	Sound and pressure *n* (%)
Ambulance Services	882 (56.1)	257 (16.3)	116 (7.3)	-	-	-
Fire and Rescue	307 (43.5)	-	83 (11.7)	120 (17)	-	-
Rural Fire	-	30 (14.9)	28 (13.9)	37 (18.4)	-	-
State Emergency Services	36 (18.2)	-	55 (27.9)	48 (24.3)	-	-
Communications Centre	6 (20)	-	-	-	6 (20)	6 (20)
Total of all services (%)	1231 (45)	287 (10.6)	282 (10.4)	205 (7)	6 (0.2)	6 (0.2)

(n = total injuries for previous 10-years for that population, (%) is for overall percentage of injuries reported to that population)

## Discussion

The aim of this study was to identify the most common injury types, locations, and mechanisms among the various EFR populations comprising an Australian State Emergency Services Agency. The results from this study suggest that the SES sustained the highest number of injuries with Fire and Rescue sustaining the least after adjusting for exposure (2054.2 vs. 183.7 FTE, respectively). Among all agencies, the low back was the most common location of injury, followed by the wrist and hand, while the shoulder was the third most common. The nature of injuries sustained throughout all populations were predominately comprised of soft tissue followed by contusions, bruises or hematomas, and superficial injuries. Meanwhile, the most common mechanism of injury between all agencies was due to body stressing, followed by being hit with a moving object, and hitting objects with the body. The findings support the hypothesis, demonstrating differences in injury rates and mechanisms of injury, but no differences in nature of injury, between departments. Interestingly, contrary to the original hypothesis, the low back and hand/wrist were the most common injured body sites between all departments.

### Ambulance

The findings within the present study identified that Ambulance Services have high rates of injury that are similar to other reports within Australia [6] and consistent with injury types found globally [1]. The most common injury location in this investigation within the Ambulance Service was the lower back, hand/wrist, and shoulder which is in line with investigations of other Australian ambulance services whereby the most commonly injured sites were the upper limb and trunk [1]. The prevalence of this location of injury is thought to be due to the increased demand placed on the body through stretcher manipulations and patient handling [11] which, in this report, resulted in predominantly soft tissue injuries.

High rates of soft tissue injuries identified in this study align with previous Australian [1] and US ambulance service studies [1, 23]. Likewise, the most common mechanism of injury in the Ambulance service was reported to be body stressing, due to lifting, carrying, or putting down objects, in agreement with another other investigation of ambulance services in Australia [5] and the USA [4]. Such high injury rates within Ambulance Services, may be due to many paramedics self-reporting low levels of fitness [24], as their primary requirement is their clinical skills, not physical abilities. Research has shown that those who are less fit are more likely to be injured in first responder populations [25, 26], possibly explaining the high injury rate amongst ambulance personnel compared to Fire and Rescue, who are reportedly more fit. For example, when measured with VO2 Max, a measure of the cardiorespiratory system during physical exertion [27], paramedics are less fit than firefighters (36 VO2max.kg-1 vs. 46 VO2max.kg-1, respectively) [28, 29]. What’s more concerning is that paramedic students report VO2 Max levels nearing that of firefighting students [28, 30]. Taken together, as paramedics get ready for the workforce their fitness decreases.

### Fire and rescue

A slightly different injury profile was found within Fire and Rescue personnel, the hand or wrist, the knee, and the lower back being the most commonly injured body parts. Although Fire and Rescue possessed the greatest number of full-time workers, they sustained the lowest rate of injuries when compared to Ambulance Services (AS: FR 0.32 [0.30–0.35]) along with the least number of injuries when reported per 1000 FTE (183.7). Other investigations in Australia have found similar results with lower rates of injury for Fire and Rescue when compared to injuries within the Ambulance services (101 vs. 150 per 1000 workers covered, respectively) [6]. This is likely due to the study reporting on the firefighting population across Australia rather than the specific population from one state. A novel finding of this study is the high number of injuries in firefighters to the hand and wrist. In a systematic review of firefighter injuries by Orr et al., [2], the more commonly reported injury sites were the lower extremity and lower back, with hand and wrist injuries only being reported by one of the studies. This difference may be due to the injury mechanisms reported in each study, where the present study identified body stressing, Orr et al. [2] reported slips trips and falls. Another explanation is that the review by Orr et al., [2], included 1,723,672 firefighters while in the present study there were only 3,888 firefighters.

### Rural fire

Research often fails to discriminate between the different branches within the fire service, such as Fire and Rescue and Rural Fire Service. This may be detrimental to understanding and mitigating injuries within each branch. Findings from this study identify that there are significant differences in the injury rates between the groups, with Rural Fire incident rate being 7-times higher than Fire and Rescue, despite Fire and Rescue’s 10-year headcount being 24-times that of Rural Fire. Within the Rural Fire service, the most common injured body sites were the hand/wrist, the head/face, and the foot/ankle. Within Rural Fire, while this study identified the upper extremity as the most common injured body location, other reports are more commonly the lower extremity [31–33]. One difference in the findings may be the total number of injuries. For example, Britton et al., [31] reported 260 injuries per annum with Moody et al., [33] reporting 90.6 while in the present study there was 20.1. Another difference may be that in the study by Garcia-Heras et al., [32] injury data was collected retrospectively through self-reports online.

In line with Fire and Rescue, Rural Fire sustained soft tissue injuries more commonly followed by superficial, contusions, and bruising. Within Rural Fire service, slips, trips, and falls were the most common injury mechanism impacting the hand/wrist the most. Findings align with previous studies of Wildland firefighters by Britton et al., [31] and Moody et al., [33] that slips, trips, and falls were the most common injury mechanism. However, within the same studies, findings are contradicted by the most common injury location being the lower limb [31, 33]. A reason for the difference may be that many included in the present study are predominantly volunteers [34, 35] with volunteer firefighters reporting higher rates of fatigue than career firefighters [36, 37] with fatigue being known to increase the risk of injury [38]. The increased risk of fatigue may be due to the volunteers already having full time jobs outside of their volunteer hours [36]. Additionally, volunteer firefighters tend to have less balance and stability [39] and can be overall less fit [40] than their career counter parts, thus placing them at greater risk of injury.

### State emergency services

Injury reports within volunteer groups such as SES are rare, making this investigation novel. Given the similar environment in which they operate, the injury profile of SES workers showed that they sustained mostly hand/wrist injuries followed by injuries to the ankle/foot and the head/face. The injuries were mostly comprised of soft tissue origin caused by hitting objects with the body. Despite the similar injury profile to the other services, the injury rate within the State SES is of note. At a rate of 2054.2 injuries per 1000 FTE for the 10-years, SES personnel is at a greater risk compared to Ambulance Services, which has been considered one of the most dangerous occupations in Australia [5]. SES are a volunteer-based occupation from all backgrounds with little age, health, or fitness requirements required for service [34]. In addition, the higher injury rates may be a result of the various tasks that this unique population must engage in.

The SES often utilize up to 13 distinctive skill sets. These are: air search, boat operations, chainsaw operations, firefighting air base support, general rescue, in-water technician, land-based swiftwater, land search and rescue, off-road driving, road crash rescue, storm damage, urban search and rescue, and vertical rescue [14]. Despite their unique requirements, these skill sets share a common task: carrying a stretcher [14]. This task has been identified by SES [14] and ambulance service personnel [11] as the most physically demanding task, which may explain the high number of upper limb injuries identified in these two services. Due to an increased utilisation of the volunteer SES within Australia, some organisations are requiring workers to undergo a Fit-for-Task program. This program assesses workers’ physical capabilities to ensure that workers are able to meet physical requirements of the job, and, in turn, reduce risk of injury [14]. It is proposed that deploying only those individuals who meet the physical capability requirements will benefit the community, organization, and the individual while minimizing risk of injury [14].

It is important to note that while the injury numbers are high within the present study, they may not represent the totality of the injuries in those who volunteer. A recent systematic review [41] of 20 studies identified several themes explaining why under-reporting occurs, such as fear of injury, effort involved in reporting, injuries being seen as part of the job, and distrust of reporting consequences [41]. The findings imply that underreporting might not only be caused by the injury but also be linked to people’s attitudes towards the work environment.

### Communications centre

One of the more unique groups in this study were the members of the Communications Centre, who sustained far fewer total injuries than every other population due to the lower number of workers. However, when expressed per 1000 FTE, the injury rate within the Communications Centre (753.7 per 1000 FTE) was far greater than both the Ambulance Service (566.3 per 1000 FTE) and Fire and Rescue service (183.7 per 1000 FTE). In a similar manner to data pertaining to the SES populations, there is a paucity of research on injuries within personnel working within Communications Centres. Interestingly, the ear was the most commonly injured location due to deafness. The deafness sustained was mainly due to a single sudden sound called acoustic shock [42]. Acoustic shock incidents are due to unexpected loud noises on telephone headsets usually caused by a sudden rise in decibels from the transmission system or from the customer end [42]. Decibels, a unit of sound energy, can also increase depending on the number of people working in a room [43]. The findings in the present study align with current research of 79 call centre operators where 98% reported at least one auditory symptom including ear pain, difficulty hearing, or hearing impairment due to loud noise episodes [19]. Furthermore, in a survey of 2,130 call centre operators, 71% experienced hearing fatigue at the end of the day while 28% reported permanent hearing concerns [18]. Factors that may decrease the chances of acoustic shock are changing ear cushions regularly and regulating decibel values [44]. A more modern way is to use a visual decibel reader visible to employees to monitor live readings of the room [43]. Other injuries within the Communications Centre included soft tissue injuries to both the hand, fingers, and low back, with an interesting finding of poisoning and toxic effects of substances being the third most common nature of injury. The poisoning and toxic effects were classified as Code 61 within the TOOCS which are from a single contact with chemical or substance [21]. Code 61 injuries could be from multiple sources, including immediate allergic reactions to a substance, splash with acid, caustic or corrosive substances in the eyes, contact dermatitis, or swallowing chemical substances [21]. Unfortunately, further details identifying which source of Code 61 injuries were responsible for the reported injuries in the Communications Centre were unavailable.

### Age

Across all services, the age groups most likely to suffer an injury in this study were those aged between 45 and 49 years old followed by ages 40–44 and 50–54. The results may be attributed to age-related reductions in upper and lower body strength, flexibility, and trunk strength for paramedics which may being to decline at 40 years of age [45]. These findings are similar to other Australian studies of non-fatal injury claims for ambulance service members who found the most injuries between the ages of 45–54 [1] and in those over the age of 40 in US firefighters [46]. Other US based studies have found different results with more injuries in those aged 30–39 years [8]. In South Korean firefighters and ambulance services, younger workers were more likely to be injured than older ones [7]. One of the reasons why the present study found age groups 45–49 more likely to be injured than previous studies may be that in Australia, the average volunteering age is 40–54 [47]. As previously outlined, 36% of all Emergency Service Agency’s staff are volunteers, with the majority of these coming from SES and Rural Fire [34, 35, 48]. Not surprisingly, these services reported the highest injury rates. Another more subtle reason is that many physical injuries may be cumulative in nature and due to repeated exposures over time [49], putting older personnel at greater risk of injury.

### Sex

Male personnel were also more likely to be injured than female personnel across all services within this study. However, males comprised 79% of the total workforce and 65% of the total injuries. The findings are in contrast to other reports in the US ambulance service where females injury rates are twice that of men while still only accounting for 33% of the total population [23]. In Australia, females make up 5% of all firefighters and, compared to males, report less injury claims [6]. In contrast, females within the Ambulance service report higher injury claims than males [6]. A potential reason may be that, in the US, female firefighters have the same occupational duties as men but suffer many injuries due to ill-fitting protective equipment [50]. Within ambulance services, females are reported to have lower levels of core and upper body strength than male paramedics but must engage in the same physical duties [45] and comprise up to 35% of all paramedics in Australia [6] which can possibly explain the high rate of injury claims.

## Injury mitigation strategies

Given their multifactorial nature, combating injury and fatigue is difficult [51]. Encouraging regular exercise both on- and off-shift to reduce levels of fatigue, and to increase or maintain general fitness levels [38, 45, 52] may be a potential strategy to reduce injury. Regular exercise programs for firefighters assisted in reducing injury rates [53, 54], enhanced job performance [55], and was potentially protective against physical overload [56]. Further benefits observed in firefighters who exercised regularly included increased aerobic fitness levels [54, 56], strength [54, 56], power [54], endurance [54], and decrease body fat percentage [54]. To further highlight the importance of physical capabilities within this population, when fatigued, firefighters who exercised regularly were able to complete simulated fire ground testing faster than those that did not regularly exercise [52].

While the relationship between injury and fitness levels are well documented in firefighters [57–59], the ambulance populations is underrepresented. Armstrong et al. [60] demonstrated the effectiveness of training programs in improving ambulance personnel readiness for duty. Ambulance personnel that adhered to their strength and conditioning program for 4-weeks, improved their lower body power, grip strength, and completed the physical employment standards testing faster than those not in the program. Furthermore, regular exercise may be more advantageous to female ambulance officers as they have been reported to have decreased lower and upper body strength compared to males while having to do similar physical demanding task [45]. Physical fitness programs specific to female EFR should emphasize total body strength and power [61] as they have higher injury rates [61]. Further research should explore how sex-specific training programs can help to mitigate injury risk.

Within the present study, as age was a factor for increased injury risk, those that are older EFR should focus on increasing cardiorespiratory fitness, upper body strength, and muscular endurance as each is related to have a significant effect in firefighter related tasks [62]. Furthermore, both ambulances [45] and firefighters [53] often show older age with a decrease in strength. Occupationally, fitter firefighters perform significantly better even as they age [62].

## Strengths and limitations

A strength of this study is the novel populations comprising the SES and the Communication Centre workers. Data pertaining to injury is currently lacking in both populations. While SES are specific to Australia, similar groups exist worldwide, such as the ARC in the USA, with limited injury data reported. The Global Burden of Disease study from 2019 [63] discusses that, although injuries may differ by region, the burden is universal and affects health, well-being, and the economy. This makes country-level injury data essential for developing global injury prevention strategies.

Furthermore, injury data collected from the Ambulance Service contributes significantly to this area’s expanding body of research. These findings provide further evidence that ambulance workers experience a notably higher rate of injuries compared to other emergency responders. This disparity underscores the need for targeted safety measures and policy interventions to better protect ambulance personnel.

This study is limited by the data due to the TOOCS injury data collection not requiring the time of day in which the injury occurred. Recent research has highlighted that firefighters and ambulance personnel that were consistently lacking in sleep were more prone to cognitive and physical fatigue and injury [38], however the lack of detail in the data make drawing this conclusion difficult in this study. Similarly, within the TOOCS system, there is no category to state whether the participant believes fatigue was a factor in their injury as fatigue has been implicated as to the reason for injury in EFR [64]. Furthermore, injury severity was not captured within this system, nor how much time was lost due to an injury. The TOOCS system only enables the user to enter time loss injuries as ‘minor- less than 1 hour lost’, ‘less than 1 day lost’, or ‘1 day or more lost’ [21], which fails to differentiate between an injury which leads to 1 day lost compared to multiple months. For example, if a person was seriously injured and was off work for multiple weeks it would be classified as the same time loss as someone off work for a single day.

## Conclusions and practical applications

Within this Emergency Services organisation, when adjusting for exposure, the SES volunteers were the most commonly injured. Injury rates differed within fire services, and subtle differences in location, nature, and mechanism of injury were found across services. Injury reduction programs need to be tailored to the unique injury profile of each service within the overall organisation. Potential short term injury risk reduction strategies include exercise to increase total body strength, muscular endurance, and cardiovascular fitness. Future research needs to identify the effect of fatigue on injury within emergency service workers.

## Data Availability

The data that support the findings of this study are available from Emergency Services Agency but restrictions apply to the availability of these data, which were used under license for the current study, and so are not publicly available. Data are however available from the authors upon reasonable request and with permission of Australian State Emergency Services Agency.
